# A multicenter study on practices and related factors of traditional medicinal plant use during pregnancy among women receiving antenatal care in East Gojjam Zone, Northwest Ethiopia

**DOI:** 10.3389/fpubh.2023.1035915

**Published:** 2023-04-17

**Authors:** Amsalu Taye Wondemagegn, Girma Seyoum

**Affiliations:** ^1^Department of Biomedical Sciences, School of Medicine, Debre Markos University, Debre Markos, Ethiopia; ^2^Department of Anatomy, College of Health Sciences, Addis Ababa University, Addis Ababa, Ethiopia

**Keywords:** traditional medicines, herbal medicines, related factors, antenatal care, Ethiopia

## Abstract

**Background:**

Little is known about the practice of traditional medicinal plant use, especially during pregnancy in Ethiopia. Moreover, there has been no previous studies conducted on practices and related factors of medicinal plant use among pregnant women in Gojjam, northwest Ethiopia.

**Methods:**

A multicentered facility-based cross-sectional study was conducted from July 1 to 30 2021. A total of 423 pregnant mothers receiving antenatal care were included in this study. To recruit study participants, multistage sampling techniques were used. Data were collected using a semi-structured interviewer-administered questionnaire. SPSS version 20.0 statistical package was used for statistical analysis. Univariable and multivariable logistic regression analysis was performed to identify factors related to the medicinal plants' utilization status in pregnant mothers. The study results were presented in both descriptive statistics (percents, tables, graphs, mean, and dispersion measurements like standard deviation) and inferential statistics (odds ratio).

**Results:**

The magnitude of traditional medicinal plants' utilization during pregnancy was 47.7% (95%CI: 42.8–52.8%). Pregnant mothers residing in rural areas [Adjusted Odds Ratio (AOR) = 3.13; 95% Confidence Interval (CI):1.53, 6.41], who are illiterate (AOR = 2.99; 95%CI:1.097, 8.17), have illiterate husbands (AOR = 3.08; 95%CI:1.29, 7.33), married farmers (AOR = 4.92; 95%CI:1.87, 12.94), married merchants (AOR = 0.27; 95%CI:0.09, 0.78), have a divorced and widowed marital status (AOR = 3.93; 95%CI:1.25, 12.395), have low antenatal care visits (AOR = 4.76; 95%CI:1.93, 11.74), substance use history (AOR = 7.21; 95%CI:3.49, 14.9), and used medicinal plants in previous pregnancy (AOR = 4.06; 95%CI:2.03, 8.13) had statistically significant association with medicinal plant use during current pregnancy.

**Conclusions:**

The present study revealed that a relatively large number of mothers used medicinal plants of various types during their current pregnancy. Area of residence, maternal educational status, husband's education level, husband's occupation status, marital status, number of antenatal care visits, use of medicinal plants in previous pregnancies, and substance use history were among the factors which were significantly associated with the use of traditional medicinal plants during the current pregnancy. Overall, the current finding provides scientific evidence useful for health sector leaders and healthcare professionals on the utilization of unprescribed medicinal plants during pregnancy and the factors associated with the utilization of the plants. Hence, they may consider creating awareness and providing advice on the careful use of unprescribed medicinal plants among pregnant mothers, especially those residing in rural areas, who are illiterate, who have divorced and widowed marital status, and who have a previous history of herbal and substance use. This is because using traditional medicines without prior discussion with a healthcare expert may harm pregnant mothers and their unborn child, as the safety of the utilized plants in the current study area is not scientifically proven. Prospective studies which need to confirm the safety of the plants used are recommended mainly in the present study area.

## Background

Traditional medicinal plant use is a medical practice that has existed in human societies since before the application of modern science to health and, thus, has an extended history (1). According to the World Health Organization (WHO) (2), traditional medicines can be defined as the sum total of the knowledge, skill, and practices based on the theories, beliefs, and experiences indigenous to different cultures, whether justifiable or not, and can be used in the maintenance of health as well as in the prevention, diagnosis, improvement, or treatment of physical and mental illness (3).

Traditional medication involves using herbal/plant medicines, animal parts, and mineral materials (4). Herbal medicines include herbs and herbal materials in their crude state as well as herbal preparations and finished herbal products that contain as active ingredients aerial or underground parts of plants or other plant materials or combinations (2) and are used mainly for the prevention and treatment of diseases (5).

Natural herbs have been taken as food or medicine to treat different diseases and for the general population to promote good health for centuries (6, 7).

Existing published evidence revealed that traditional herbal medicine use during pregnancy ranges from 1 to 87%, worldwide (8). Published studies revealed that the magnitude of herbal medicine utilization during pregnancy ranges between 1 and 60% in developed nations (9).

According to published evidence, the magnitude of herbal use among women during pregnancy in the United States (US) ranged from nearly 4–45% (10–13). A study in Saudi Arabia reported a 94% prevalence use of herbal medicines for therapeutic purposes among adult residents (14). Another study in Saudi Arabia (15) showed a 33% overall use of herbal medicines during pregnancy for different therapeutic purposes.

The WHO reported that about 80% of the African population use herbal medicine in any of its forms (16). Existing published findings showed that abundant use of herbal medicines in Africa and Asia may be related to the people's perception of lower cost, accessibility, perceived safety and efficacy, cultural significance, as well as a patient distrust of modern medical care (14, 17).

Studies done in different African countries reported that the prevalence of medicinal plants' utilization during pregnancy ranged between 12 and 80% (18–23). According to existing evidence, traditional medicinal plant use during pregnancy in Ethiopia has been reported by only a few studies in a few study areas. Accordingly, a study conducted in Arbaminch, the southern part of Ethiopia, found that 48.5% of mothers use herbal medicinal plants while pregnant (24). A study conducted in Nekemte, a western part of Ethiopia, reported that 69.8% of pregnant women used herbal medicine during pregnancy (25). A facility-based study conducted in Gondar, northwest Ethiopia, reported the magnitude of herbal medicine use during pregnancy was 48.6% and the most commonly utilized herbal medicines during pregnancy were garlic (19%) and ginger (40.7%) (26).

Despite the prevalent use of natural traditional herbal products by pregnant women, there is very little published evidence regarding the safety and efficacy of natural traditional medicinal products during pregnancy and lactation (27).

Safety worries related to herbal medicine use have started to arise. Pregnant women utilize many herbal types during pregnancy to relieve issues such as morning sickness and nausea (28). Herbal medicine use even raises a particular concern due to the fact that consumers have little way of knowing if a product will do what the label claims and how safe the product may be. Existing literature showed that the use of herbs during pregnancy has been associated with potential harm to the growing fetus (29, 30).

Accordingly, the existing few studies (31–33) done on herbal medicines found using herbs during pregnancy resulted in some adverse maternity-related outcomes such as premature birth, damage to oocytes, and iron absorption inhibition. On the contrary, other study results found no adverse pregnancy-related outcomes (34, 35). In addition, herbs may contain substances that can cause miscarriage, premature birth, uterine contractions, or injury to the fetus (19).

As reviewed from existing literature, a recent systematic review done using studies conducted in Africa found that herbal medicine use during pregnancy is significantly associated with a lower educational level, increased age, being married, low socioeconomic status, lower educational level of the husband, poor pregnancy outcomes, herbal medicine use in prior pregnancies, the perception that medicinal plants are effective, large family size, self-employment, unemployment, and rural residence (36). In the same way, existing literature showed that socio-demographic variables such as religion, educational status, economic status, parity, area of residence, and previous history of herbal medicines used for other conditions has been associated with traditional herbal medicine use among pregnant women in sub- Saharan Africa (23, 26).

Several studies have been conducted in different countries on traditional herbal medicine use among pregnant women, but there is a scarcity of information on this practice in Ethiopia, especially using a multicenter-based approach. To the researcher's knowledge, there is no abundant multicentered-based scientific evidence of traditional medicine use and associated factors during pregnancy in Ethiopia. More importantly, there is no previous attempt in the current study area, the East Gojjam Zone in particular, regarding traditional medicinal plant use and associated factors during pregnancy. Thus, the current study attempts to fill this knowledge gap. Therefore, the present study aimed to determine practice level and related factors of traditional medicinal plants use during pregnancy among women attending antenatal care in East Gojjam Zone, Northwest Ethiopia.

Knowing the practice status of herbal medicine among pregnant women would enable healthcare planners and policymakers, healthcare professionals, other regulatory bodies, and health educators to work on issues to address the irregulated use of herbal preparations among these special risky groups.

## Methods

### Study design, setting, and population

A multicentered facility-based cross-sectional study was conducted by using quantitative research methods. The study was conducted in selected east Gojjam zone health institutions Debre Markos town is the capital of the East Gojjam zone. The town is found to the east of the Amhara region, about 256 kilometers from Bihar Dar [capita town of Amhara region] and 299 kilometers from Addis Ababa [capital town of Ethiopia]. The study was conducted from July 1 to 30 2021. All permanent resident pregnant women who were receiving antenatal care follow-up in selected health institutions of east Gojjam zone and who were not seriously sick at time of data collection were the study population.

### Sample size determination and sampling procedures

The sample size was calculated using EPI INFO version 3.5 based on the assumption of 95% confidence interval, 5% margin of error, and using the traditional medicine use proportion during pregnancy which is 48.6% according to a previous study (26). The calculated sample size was 384 and the final sample size after adding 10% for non-response was 423. To recruit study participants, firstly, four health institutions, namely Debre Markos Comprehensive Specialized Hospital, Gozamen Health Center, Dejen Health Center, and Bichena Health Center, were selected using the lottery method. Next, all pregnant women visiting the selected health institutions for antenatal care and who fulfilled the inclusion criteria during the study period were selected systematically until the required sample size was fulfilled based on proportional allocation.

Study variables were traditional medicinal plants use status; socio demographic characteristics like age, residence, religion, educational level, education status of respondents' husbands, occupation status, occupation of respondents' husband, house hold income level, marital status, family types, and housing status; and pregnancy-related variables like gravidity, parity, antenatal care visits, history of substance use, previous use of traditional medicines, and health conditions during current pregnancy.

#### Data collection

The data collection questionnaire for the interview was developed by reviewing different literatures. The questionnaire was prepared in English and translated into the local language of Amharic and back-translated to English to maintain consistency. Data was collected through an interviewer-administered questionnaire. The questionnaires comprised demographic and pregnancy-related characteristics and also detailed information about herbal use both before and during pregnancy. In the parts of the questionnaire regarding herbal medicine use, both closed and open-ended questions were used to identify the type of herbal medicines used during pregnancy in the current study area. In addition, the reasons for herbal medicines' use, the administration timing, frequency, administration route, perceived effectiveness, side effects, and whether the women disclosed herbal medicine use to their health care providers were questioned. Five bachelors of science pharmacy professionals and two bachelors of science public health professionals were used for data collection and supervision, respectively, after 3 days of intensive training. The provision of 3-day intensive training to data collectors and supervisors concentrated on the aim of the study, administration of the questionnaire, survey instruments, and ethical considerations.

### Data processing and analysis

Each questionnaire was given a code and was entered in to EpiData version 3.1 statistical package and was then exported to SPSS version 20.0 statistical package for statistical analysis. Data cleaning and editing was made before analysis. The result of the study was presented in both descriptive statistics (percents, tables, graphs, mean, and dispersion measurements like standard deviation) and inferential statistics (odds ratio). Which means that before establishing association b/n dependent and independent variables; the multi-collinearity among independent variables were checked. Binary logistic regression was used to calculate the univariate and multivariate crude and adjusted odds ratio, respectively, and to identify independent predictors of dependent variables. In the multi-variate logistic regressions model, only those variables which were associated with the dependent variable with *p*-value ≤ 0.2 in univariate analysis were considered biologically important and “not collinear” was entered. Statistical significance was declared at *p*-value <0.05.

### Ethical considerations

The proposal was approved by Debre Markos university, school of medicine ethical review committee on June 28, 2021 with approval number M/R/CS/61/03/21. Permission was also obtained from the concerned bodies of East Gojjam zone. To protect confidentiality, no personal identifiers were recorded in the questionnaire and the recorded data was not accessed by a third party. Verbal informed consent to participate in the study was obtained from study participants.

## Results

### Sociodemographic and economic characteristics of study participants

Out of 423 planned study participants, 390 participated in the study, making a response rate of 92.2%. The mean age of the study participants was 30.8 years with standard deviation of ±4.0, and a majority (79.2%) of the study participants were within the age group of 25–35 years. A majority of the participants were living in a rural area (59.2%), had tertiary-level education, had illiterate and farmer husbands, were married, had an average monthly income of 2,000–4,000 Birr, were orthodox religion followers, had nuclear family types, and owned their own homes (Table 1).

**Table 1:** Sociodemographic and economic characteristics of the study participants by their medicinal plant use status in East Gojjam Zone, Northwest Ethiopia, June 2021 (*N* = 390).

**Variables**	**Coding categories**	**Traditional medicinal plants use status**	
		**Non-users, frequency (%)**	**Users, frequency (%)**
Age group of the study participants	< 25 years	15 (7.4%)	10 (5.4%)
	25–35 years	161 (78.9%)	148 (79.6%)
	>35 years	28 (13.7%)	28 (15.1%)
Participant's area of residence	Urban	107 (52.5%)	52 (28%)
	Rural	97 (47.5%)	134 (72%)
Educational status	No formal education	27 (13.2%)	65 (34.9%)
	Primary education	61 (29.9%)	40 (21.5%)
	Secondary education	52 (25.5%)	33 (17.7%)
	College/university education	64 (31.4%)	48 (25.8%)
Husband's educational level	No formal education	60 (31.6%)	119 (64%)
	Primary education	27 (14.2%)	15 (8.1%)
	Secondary education	15 (7.9%)	10 (5.4%)
	College/university education	88 (46.3%)	42 (22.6%)
Participant's occupation status	House wife	36 (17.6%)	35 (18.8%)
	Farmer	10 (4.9%)	67 (36%)
	Government employment	79 (38.7%)	17 (9.1%)
	Merchant	43 (21.1%)	27 (14.5%)
	Daily laborer	36 (17.6%)	40 (21.5%)
Husband's occupation status	Farmer	16 (8.4%)	105 (56.5%)
	Government employment	69 (36.3%)	18 (9.7%)
	Merchant	56 (29.5%)	18 (9.7%)
	Daily laborer	49 (25.8%)	45 (24.2%)
Participant's marital status	Not married	58 (28.4%)	61 (32.8%)
	Married	120 (58.8%)	90 (48.4%)
	Others/divorced and widowed	26 (12.7%)	35 (18.8%)
Average monthly household income	< 2,000 birr	65 (31.9%)	58 (31.2%)
	2,000–4,000 birr	60 (29.4%)	92 (49.5%)
	>4,000 birr	79 (38.7%)	36 (19.4%)
Religion of the study participants	Orthodox	168 (82.4%)	153 (82.3%)
	Muslim	36 (17.6%)	33 (17.7%)
Family types of the participants	Nuclear	188 (92.2%)	56 (30.1%)
	Extended	16 (7.8%)	130 (69.9%)
Housing conditions of participants	Rental	95 (46.6%)	75 (40.3%)
	Own house	109 (53.4%)	111 (59.7%)

### Pregnancy-related characteristics of the study participants

Out of 390 participants, 280 (71.8%) were in the 1st trimester of pregnancy. A majority of the study participants were para 2, gravida 3, on their first antenatal care visit, and sometimes used substances (Table 2). Of the 390 participants, 213 (54.6%) had experienced medical problems during their current pregnancy and 96 (45.1%) treated the condition using medicinal plants, 27 (12.7%) with conventional medicines, and 90 (42.3%) both.

**Table 2:** Revealed pregnancy-related characteristics of the study participants in East Gojjam Zone, Northwest Ethiopia, 2021 (*N* = 390).

**Variables**	**Coding categories**	**Traditional medicinal plant use status**	
		**Non-users, frequency (%)**	**Users, frequency (%)**
Duration/months of pregnancy at time of interview (*N* = 390)	1st trimester	128 (62.7%)	152 (81.7%)
	2nd trimester	74 (36.3%)	14 (7.5%)
	3rd trimester	2 (1%)	20 (10.8%)
Parity (*N* = 390)	Having 1 children	36 (17.6%)	36 (19.4%)
	Having 2 children	128 (62.7%)	74 (39.8%)
	Having ≥3 children	40 (19.6%)	76 (40.9%)
Gravidity [number of total pregnancy (*N* = 390)]	2 pregnancies	19 (9.3%)	35 (18.8%)
	3 pregnancies	137 (67.2%)	64 (34.4%)
	≥4 pregnancies	48 (23.5%)	87 (46.8%)
Number of Ante Natal Care (ANC) visits (*N* = 390)	1st ANC visit	146 (71.6%)	154 (82.8%)
	2nd ANC visit	58 (28.4%)	32 (17.2%)
Substances use (*N* = 390)	Not at all	139 (68.1%)	46 (24.7%)
	Sometimes	65 (31.9%)	140 (75.3%)
Presence of medical problems during current pregnancy (*N* = 390)	Yes	95 (46.6%)	118 (63.4%)
	No	109 (53.4%)	68 (36.6%)

### Patterns of traditional medicinal plant utilization among pregnant women in East Gojjam Zone, Northwest Ethiopia

**Figure 1:**
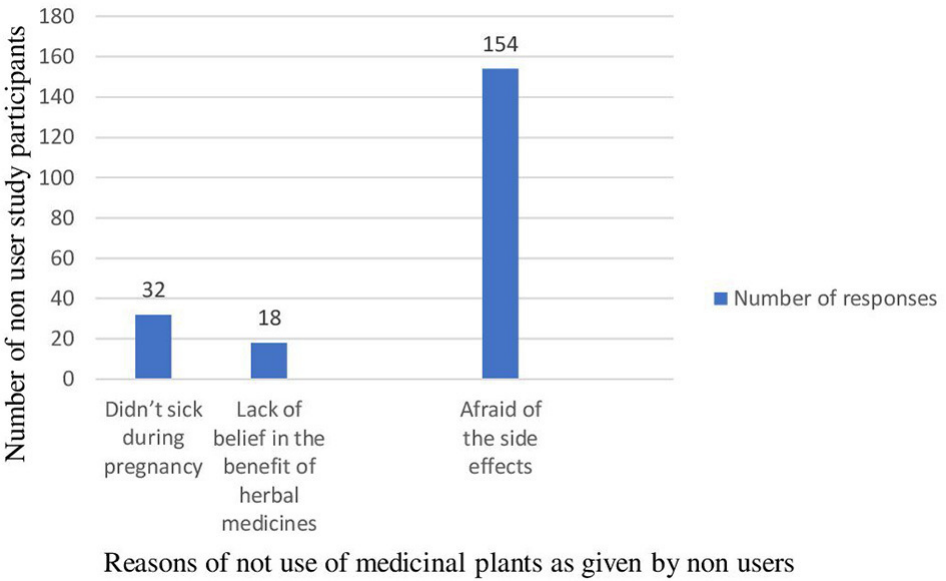
Reasons for not using medicinal plants among non-users in East Gojjam Zone, Northwest Ethiopia, 2021 (*N* = 204).

**Figure 2:**
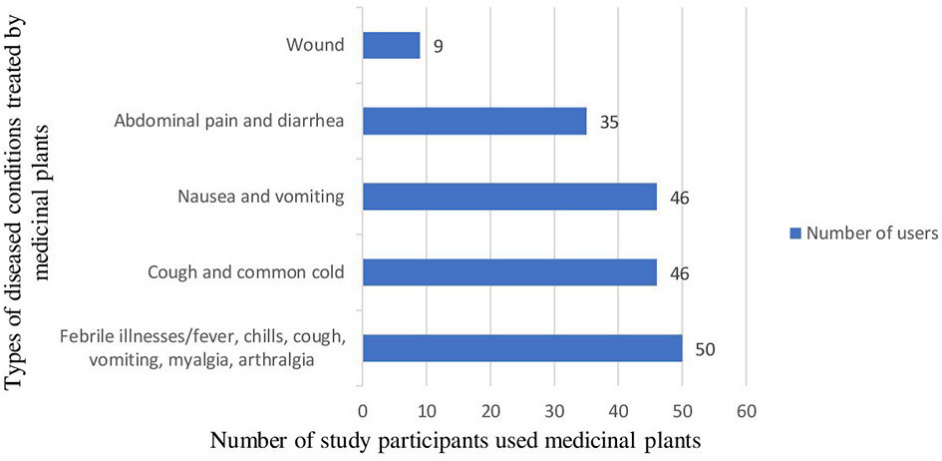
Diseased conditions treated by the study participants using medicinal plants in East Gojjam Zone, Northwest Ethiopia, 2021 (*N* = 186).

Among the study participants 186 [47.7% (95% CI: 42.8–52.8%)] had practiced traditional medicinal plant use. A majority (87.1%) of the study participants also used traditional medicinal plants during the 2nd and 3rd months of pregnancy. The major reasons reported for the use of herbal medicines during pregnancy were their perception them being of safer (34.4%) and more effective (32.3%) than conventional medicine (Table 3). On the other hand, the major perceived reason reported for not taking medicinal plants during current pregnancy by the non-user study participants were fear of the side effects (Figure 1). The most common medicinal plants used were Nech shinkurt (24.7%) and Damakase (19.4%) (Table 4). The major disease conditions treated by the plants were febrile illnesses (50), cough and common cold (46), and nausea and vomiting (46) (Figure 2).

**Table 3:** Patterns of traditional medicinal plant utilization by the study participants in east Gojjam Zone, Northwest Ethiopia, 2021 (*N* = 390).

**Variables**	**Coding categories**	**Frequency (%)**
History of use of medicinal plants in past pregnancies (*N* = 390)	Yes	188 (48.2%)
	No	202 (51.8%)
Traditional medicinal plant use during current pregnancy (*N* = 390)	Yes	186 (47.7)
	No	204 (52.3)
Trimester of pregnancy medicinal plants were taken (*N* = 186)	1st trimester	162 (87.1%)
	2nd trimester	24 (12.9%)
Frequency of medicinal plants used (*N* = 186)	Only once	34 (18.3%)
	Occasionally	132 (71%)
	Weekly	13 (7%)
	Twice or more per week	7 (3.8%)
Who recommended the use of medicinal plants (*N* = 186)	My own idea	50 (26.9%)
	Family	69 (37.1%)
	Friends or neighbors	41 (22%)
	Traditional healers	26 (14%)
Sources/places used to obtain medicinal plants (*N* = 186)	Self-preparation	50 (26.9%)
	Traditional healers or herbalists	26 (14%)
	Family and/or friends	110 (59.1%)
Reasons for use of medicinal plants during pregnancy (*N* = 186)	Herbal medicines are more effective than conventional medicines	60 (32.3%)
	Herbal medicines are safe in pregnancy	64 (34.4%)
	Herbal medicines are much cheaper than conventional medicines	22 (11.8%)
	Mistrust of conventional medicines	20 (10.8%)
	Herbal medicines are more accessible than conventional medicines	20 (10.8%)
Inform health professionals about medicinal plants use during pregnancy (*N* = 186)	Yes	27 (14.5%)
	No	159 (85.5%)
Reasons of non-disclosure (*N* = 186)	Forgot it	26 (14%)
	It was not important	79 (42.5%)
	Afraid of health professional's response	81 (43.5%)
Adverse effects faced while using medicinal plants (*N* = 186)	Yes	43 (23.1%)
	No	143 (76.9%)
How do you rate your satisfaction level of using medicinal plants (*N* = 186)	Satisfied	108 (58.1%)
	Less satisfied	33 (17.7%)
	Not satisfied	45 (24.2%)
Reasons of not using medicinal plants ever among non-users (*N* = 204)	Didn't feel sick during pregnancy	32 (15.7%)
	Lack of belief in the benefit of herbal medicines	18 (8.8%)
	Afraid of the side effects	154 (75.5%)

**Table 4:** Types of medicinal plants utilized among the study participants in East Gojjam Zone, Northwest Ethiopia, June 2021 (*N* = 186).

**Medicinal plants type**		**Frequency (%)**	**Diseases conditions treated**
**Scientific name**	**Local name (Amharic)**		
*Allium sativum* L. (Alliaceae)	Nech Shinkurt	46 (24.7)	Cough, common cold, nausea, vomiting, abdominal pain
*Ocimum urticifolium* Roth (Lamiaceae)	Damakase	36 (19.4)	Febrile illnesses/fever, chills, cough, vomiting, myalgia, arthralgia
*Ruta chalepensis* L. (Rutaceae)	Tenaadam	22 (11.8)	Cough, common cold, stomachache/gastritis
*Lepidium sativum* L. (Brassicaceae)	Feto	16 (8.6)	Nausea, vomiting, abdominal pain, diarrhea
*Vernonia amygdalina* Del. (Asteraceae)	Girawa	15 (8.1)	Nausea, vomiting, abdominal pain, diarrhea
*Eucalyptus globulus* Labill. (Myrtaceae)	Nech bahir zaf	15 (8.1)	Cough, common cold, stomachache/gastritis
*Zehneria scabra* (Linn. F.) Sondll. (Cucurbitaceae)	Hareg ressa	14 (7.5)	Febrile illnesses/fever, chills, cough, vomiting, myalgia, arthralgia
*Nigella sativa* L. (Ranunculaceae)	Tikur-azmud	6 (3.2)	Cough and common cold
*Coffea arabica* L. (Rubiaceae)	Bunna	6 (3.2)	Wound
*Embelia schimperi* Vatke (Myrsinaceae)	Enqoqo	4 (2.2)	Nausea, vomiting, abdominal pain, diarrhea
*Plantago lanceolata* L. (Plantaginaceae)	Gorteb	3 (1.6)	Wound
*Cucumis ficifolius* A. Rich (Cucurbitaceae)	Yemidir Embuway	3 (1.6)	Cough and common cold

### Factors associated with medicinal plant use in East Gojjam Zone, Northwest Ethiopia

Univariable and multivariable logistic regression analysis result revealed that participants area of residence, education status, husbands' education level, husbands occupation status, participants marital status, average monthly households income, parity, gravidity, number of antenatal care visits, history of substance use, medicinal plants use in the previous pregnancies and experience of medical problems in the current pregnancy were significantly associated with medicinal plants utilization during current pregnancy (Table 5).

**Table 5:** Univariable and multivariable analysis results of factors associated with medicinal plant use in East Gojjam Zone, Northwest Ethiopia, 2021 (*N* = 390).

**Variables with coding categories**	**Traditional medicinal plants use status**		**COR (95% CI)**	**AOR (95% CI)**
	**Non-users, frequency (%)**	**Users, frequency (%)**		
**Age groups of study participants**				
< 25 years	15 (7.4%)	10 (5.4%)	0.67 (0.26, 1.74)	0.54 (0.10, 2.77)
25–35 years	161 (78.9%)	148 (79.6%)	0.92 (0.52, 1.62)	0.74 (0.23, 2.41)
>35 years	28 (13.7%)	28 (15.1%)	1	1
**Participant's area of residence**				
Urban	107 (52.5%)	52 (28%)	1	1
Rural	97 (47.5%)	134 (72%)	2.84 (1.86, 4.34)^**^	3.13 (1.53, 6.41)^**^
**educational status**				
No formal education	27 (13.2%)	65 (34.9%)	3.21 (1.79, 5.76)^**^	2.99 (1.09, 8.17)^*^
Primary education	61 (29.9%)	40 (21.5%)	0.87 (0.51, 1.51)	0.82 (0.32, 2.09)
Secondary education	52 (25.5%)	33 (17.7%)	0.85 (0.48, 1.50)	0.72 (0.27, 1.93)
College/university education	64 (31.4%)	48 (25.8%)	1	1
**Husband's educational level**				
No formal education	60 (31.6%)	119 (64%)	4.16 (2.57, 6.72)^**^	3.08 (1.29, 7.33)^*^
Primary education	27 (14.2%)	15 (8.1%)	1.16 (0.56, 2.42)	1.02 (0.29, 3.61)
Secondary education	15 (7.9%)	10 (5.4%)	1.39(0.58, 3.37)	0.94 (0.19, 4.41)
College/university education	88 (46.3%)	42 (22.6%)	1	1
**Husband's occupation status**				
Farmer	16 (8.4%)	105 (56.5%)	7.15 (3.68, 13.88)^**^	4.92 (1.87, 12.94)^**^
Government employment	69 (36.3%)	18 (9.7%)	0.28 (0.15, 0.55)^**^	0.37 (0.12, 1.09)
Merchant	56 (29.5%)	18 (9.7%)	0.35 (0.18, 0.68)^**^	0.27 (0.09, 0.78)^*^
Daily laborer	49 (25.8%)	45 (24.2%)	1	1
**Participant's marital status**				
Not married	58 (28.4%)	61 (32.8%)	1	1
Married	120 (58.8%)	90 (48.4%)	0.71 (0.45, 1.12)	0.69 (0.33, 1.45)
Others/divorced and widowed	26 (12.7%)	35 (18.8%)	1.28 (0.69, 2.38)	3.93 (1.25, 12.39)^*^
**Average monthly households' income**				
< 2,000 birr	65 (31.9%)	58 (31.2%)	1.96 (1.15, 3.33)^*^	1.52 (0.63, 3.67)
2,000–4,000 birr	60 (29.4%)	92 (49.5%)	3.37 (2.02, 5.61)^**^	1.89 (0.79, 4.55)
>4,000 birr	79 (38.7%)	36 (19.4%)	1	1
**Religion of the study participants**				
Orthodox	168 (82.4%)	153 (82.3%)	0.99 (0.59, 1.67)	0.76 (0.32, 1.81)
Muslim	36 (17.6%)	33 (17.7%)	1	1
**Parity**				
Having 1 children	36 (17.6%)	36 (19.4%)	1	1
Having 2 children	128 (62.7%)	74 (39.8%)	0.58 (0.34, 0.99)^*^	1.03 (0.32, 3.36)
Having ≥3 children	40 (19.6%)	76 (40.9%)	1.90 (1.04, 3.46)^*^	0.95 (0.17, 5.37)
**Gravidity**				
2 pregnancies	19 (9.3%)	35 (18.8%)	1	1
3 pregnancies	137 (67.2%)	64 (34.4%)	0.25 (0.14, 0.48)^**^	0.35 (0.09, 1.23)
≥4 pregnancies	48 (23.5%)	87 (46.8%)	0.98 (0.51, 1.90)	1.88 (0.32, 10.97)
**Number of ANC visits**				
1st ANC visit	146 (71.6%)	154 (82.8%)	1.91 (1.17, 3.11)^**^	4.76 (1.93, 11.74)^**^
2nd ANC visit	58 (28.4%)	32 (17.2%)	1	1
**History of substances use**				
Not at all	139 (68.1%)	46 (24.7%)	1	1
Sometimes	65 (31.9%)	140 (75.3%)	6.51 (4.17, 10.15)^**^	7.21 (3.49, 14.9)^**^
**Medicinal plants use in previous pregnancy**				
Yes	64 (31.4%)	124 (66.7%)	4.38 (2.86, 6.69)^**^	4.06 (2.03, 8.13)^**^
No	140 (68.6%)	62 (33.3%)	1	1
**Presence of medical problems**				
Yes	95 (46.6%)	118 (63.4%)	1.99 (1.33, 2.99)^**^	1.19 (0.59, 2.42)
No	109 (53.4%)	68 (36.6%)	1	1

* p < 0.05,

** p < 0.005, COR, crude odds ratio; AOR, adjusted odds ratio; CI, confidence interval.

The current study found that t pregnant women living in a rural area had 3 times more utilization of medicinal plants (AOR = 3.13; 95%CI: 1.53, 6.41) compared to pregnant women living in an urban area. Illiterate pregnant women had 3 times increased risk of use of medicinal plants during their current pregnancy (AOR = 2.99; 95%CI: 1.09, 8.17) compared to tertiary-level educated women. Women with illiterate husbands had 3 times the intake of traditional medicinal plants (AOR = 3.08; 95%CI: 1.29, 7.33) compared to those pregnant women married to tertiary-level educated husbands. Pregnant mothers married to farmers had 5 times increased utilization of medicinal plants (AOR = 4.92; 95%CI: 1.87, 12.94) compared to pregnant mothers with daily laborer husbands. On the other hand, pregnant mothers with government-employed husbands had a 72% decreased use of medicinal plants (COR = 0.28; 95%CI: 0.15, 0.55) and those who had merchant husbands had a 73% decreased utilization of medicinal plants (AOR = 0.27; 95%CI: 0.09, 0.78) compared to pregnant mothers with daily laborer husbands. Divorced and widowed pregnant mothers had 4 times the utilization of medicinal plant use (AOR = 3.93; 95%CI: 1.25, 12.39) compared to never married mothers. Pregnant women with an average monthly income of <2,000 birr had 2 times more utilization (COR = 1.96; 95%CI: 1.15, 3.33) and with 2,000–4,000 birr 3 times increased utilization (COR = 3.37; 95%CI: 2.02, 5.61) of medicinal plants compared to pregnant mothers with an average monthly income of >4,000 Birr. Mothers with 2 children had a 42% decreased use of plants (COR = 0.58; 95%CI: 0.34, 0.99) and mothers with 3 or more children had 1.9 times more use of medicinal plants (COR = 1.9; 95%CI: 1.04, 3.46) compared to mothers with one child. Participants with three total pregnancies had a 75% decreased use (COR = 0.25; 95%CI: 0.14, 0.48) compared to mothers with two total pregnancies. Pregnant mothers with only one antenatal care visit had 5 times increased utilization of medicinal plants (AOR = 4.76; 95%CI: 1.93, 11.74) compared to those with two visits. Pregnant mothers with a substance use history had 7 times increased use of medicinal plants (AOR = 7.21; 95%CI: 3.49, 14.9) compared to those who did not. Pregnant mothers who used medicinal plants in their previous pregnancy had 4 times increased use (AOR = 4.06; 95%CI:2.03, 8.13) of medicinal plants compared to those who did not use in their previous pregnancies. Mothers who had experienced medical health problems in their current pregnancy had 2 times higher (COR = 1.99; 95%CI: 1.33, 2.99) use of medicinal plants compared to mothers who did not experience medical problems.

## Discussion

The present study found that utilization rates of medicinal plants among pregnant mothers were 47.7%, which is higher than those in Saudi Arabia at 33% (15), in Nairobi, Kenya at 12% (23), and in Alexandria Egypt (22) at 27.3% practice, but lower than those practices in Mali, west Africa at 80% (18), in Nigeria (19) at 67.5% utilization, in Sierra Leone (20) at 62.7%, in northern Ghana (21) at 52.7%, and in Nekemte, west Ethiopia, at 69.8% (25). But the current finding is consistent with those reported in Arbaminch (48.5%) (24), in southern Ethiopia, and those practices in Gondar (48.6%) (26), northwest Ethiopia. The variability in magnitude of practices may be attributed to variations in culture, socioeconomic status, education status, perception of modern medicines, and availability and accessibility of health facilities.

The present study found that being a rural resident made a pregnant woman statistically more likely to use medicinal plants compared to those living in urban area. This finding is consistent with systematic review done in Africa (36) and the original study done in Gondar, northwest Ethiopia (26). This could be due to the accessibility and availability of health facilities, meaning mothers in urban areas may get easier access to qualified health professions when facing health problems and they also have a higher chance of seeing qualified health professionals in order to consult and obtain health information relevant to pregnancies.

The present study found that being non-educated and married to less educated husbands related to significantly increased use of medicinal plants as compared to those having high education status and married to husbands with a higher education level. The finding is consistent with previous studies (23, 26, 36). This could be due to the fact that being educated and having educated husbands may increase health-seeking behavior, which enables them to choose appropriate health care help in the case of health problems. In addition, having an educated family may mean better household income which enables women to determine access to conventional care instead of choosing medicinal plants use when ill. Lastly, being educated may enable mothers to have better skills, coping mechanisms, or strategies for realization of better health outcomes (37). In contrast to our findings, a study in Saudi Arabia (15) revealed a statistically significant use of herbal medicines by educated pregnant women as compared to less educated pregnant women.

The current study found that mothers whose husbands were farmers had significantly increased medicinal plant use and those with merchant husbands had decreased use of medicinal plants. This could be because occupation status may be associated with educational status, in that uneducated husbands may not seek healthy behaviors when his wife is sick and thus, mothers may choose taking medicinal plants when facing health problems. On the other hand, married merchant husbands may have a better income and may choose healthier modern health care when experiencing medical problems.

In the current study, mothers who were divorced or widowed had significantly increased utilization of medicinal plants, but the previous systematic review reported being married had an increased risk of utilization (36). One possible explanation would be being divorced or widowed may have an impact on household income and thus, mothers may use medicinal plants when sick due to the perception of decreased costs of medicinal plants.

In the present study, pregnant women with less antenatal care visits were more likely to utilize medicinal plants as compared to those with more visits. This could be because repeated visits to health facilities may enable mothers to have more information regarding bad practices during pregnancy.

In this study, participants who practiced utilization of substances such as alcohol, coffee, and others had increased use of medicinal plants compared to those who did not take any substances. This could be due to the fact that traditional medicinal plants can be used for treatments of substance withdrawal or relief from symptoms of relapse (38, 39).

In the present study, pregnant mothers with a history of medicinal plant use in their previous pregnancies had increased utilization of medicinal plants in the current pregnancy. This could be due to their previous behavior affecting their current practices.

In this study, the main reasons for pregnant mothers using herbal medicines during pregnancy were their perception of safety and effectiveness as compared to the use of conventional medicines. Our finding is consistent with previous reports conducted elsewhere (14, 17).

In the present study, pregnant mothers with three or more children had increased use of medicinal plants as compared to those with one child. This may be because pregnant mothers with more children may have previous practices of plant utilization and may not have experienced adverse effects in their last pregnancies. On the other hand, pregnant mothers with two children in the present study had a decreased utilization of plants during their current gestation. This may be due to mothers with two children having previous experience of utilization with adverse effects on their last pregnancies. In addition, pregnant mothers with two children who participated in the present study may be educated and may have information on the harm of using unprescribed herbal medications.

Since our study findings are based upon a cross-sectional design, it did not enable us to form a conclusion on the casual relationship. Moreover, first-time pregnant mothers were not accessed during the study period. This may be due to the fact that first-time pregnant mothers may be in fear of exposing their pregnancy status and hence may not visit the selected health institutions. Thus, the current study may lack evidence about the utilization status and the triggering factors of the utilization of medicinal plants for first-time pregnant mothers. Being a multicentered study and using both urban and rural populations for the study were the major strengths of the present study.

## Conclusion

The present study revealed that a relatively large number of mothers used medicinal plants of various types during their current pregnancy. Area of residence, maternal educational status, husband's education level, husband's occupation status, marital status, number of antenatal care visits, use of medicinal plants in the previous pregnancies, and substance use history were among the factors which were significantly associated with the use of traditional medicinal plants during the current pregnancy. Overall, the current finding provides scientific evidence useful for concerned health sector leaders and healthcare professionals on the utilization of unprescribed medicinal plants during pregnancy and the factors associated with the utilization of the plants. Hence, they may consider raising awareness and providing advice on the careful use of unprescribed medicinal plants among pregnant mothers, especially those residing in rural areas, who are illiterate, who have a divorced or widowed marital status, and who have a previous history of herbal and substance use. This is because using traditional medicines without prior discussion with a healthcare expert may harm pregnant mothers and their unborn babies, as the safety of the utilized plants in the current study area is not scientifically proven. Prospective studies which need to confirm the safety of the plants used are recommended in the present study area.

## Data availability statement

The original contributions presented in the study are included in the article/Supplementary material, further inquiries can be directed to the corresponding author.

## Ethics statement

The studies involving human participants were reviewed and approved by the Debre Markos University, School of Medicine Ethical Review Committee on June 28, 2021 with number of M/R/CS/61/03/21. Written informed consent for participation was not required for this study in accordance with the national legislation and the institutional requirements.

## Author contributions

AW was the principal investigator involved in conception and design of the study, data collection, entry, analysis, and interpretation of the data, and prepared the manuscript. GS was involved in design of the study, entry, analysis, and interpretation of data as well as manuscript preparation. All authors contributed equally to this work.

## Supplementary material

Supplementary Table S1 Tools used for this study.
