# Supplementary material for: A multicenter study on practices and related factors of traditional medicinal plant use during pregnancy among women receiving antenatal care in East Gojjam Zone, Northwest Ethiopia
Source: Front Public Health. 2023 Apr 17;11:1035915. doi: 10.3389/fpubh.2023.1035915 (PMC10149730; doi:10.3389/fpubh.2023.1035915)
Supplement: Supplementary Table S1 — Tools used for this study. [file Table_1.DOCX]

**Questionnaire for the Use of Medicinal Plants among Pregnant Women in East Gojjam zone, Northwest Ethiopia.**

Questionnaire ID__________________

| **Part I: Sociodemographic and economic characteristics of participants** | | | |
| --- | --- | --- | --- |
| **No** | **Variables** | Coding category | Remark |
| 101 | Age | ----------------------in years |  |
| 102 | Residence | 1. Urban 2. Rural |  |
| 103 | Educational status | 1. No education 2. Non-formal education 3. Primary education 4. Secondary education 5. College/University education |  |
| 104 | husband educational level | 1. No education 2. Non-formal education 3. Primary education 4. Secondary education 5. College/University education |  |
| 105 | Occupation status | 1. House wife 2. Farmer 3. Employed 4. Merchant 5. Daily laborer 6. Other specify |  |
| 106 | Occupation status of husband’s | 1. Farmer 2. Employed 3. Merchant 4. Daily laborer 5. Other specify |  |
| 107 | Marital status | 1. Never married/single 2. Currently married 3. Divorced/separated 4. Widowed |  |
| 108 | HH monthly/yearly income | ___________________ |  |
| 109 | What is your religion? | 1.Orthodox 2. Muslim  3. Protestant 4. Catholic  5. Others(specify) |  |
| 110 | What is your family type? | 1. Nuclear  2. Extended  3. Other |  |
| 111 | What are your housing conditions? | 1. Rental  2. Has own house  3. Others |  |
| Part II**: Pregnancy related variables** | | | |
| 201 | Month of your current pregnancy | __________________ |  |
| 202 | What is your parity currently |  |  |
| 203 | How many pregnancies have you carried so far? |  |  |
| 204 | How many ANC visits do you have so far? |  |  |
| 205 | Have you ever consumed any substances/alcohol, coffee, khat, cigarette/ during your pregnancy? | 1. Not at all  2. Sometimes  3. Daily  4. I use substances before I became pregnant |  |
| 206 | Are you currently taking any pharmaceutical drugs, i.e., medicines acquired from pharmacies or clinics? /  Conventional drug use | 1. yes  2. no |  |

| **Part III: Use of Herbal Medicines** | | | |
| --- | --- | --- | --- |
| 301 | Have you ever used herbal medicines or medicinal plants in past pregnancies? | 1. Yes  2. No  3. Unsure |  |
| 302 | Have you used herbal medicines before (even if not pregnant) | 1. Yes  2. No |  |
| 303 | Are you currently taking any herbal medicines or medicinal plants? | 1. Yes  2. No | If yes fill the table below |

If “Yes”, please complete the following table:

| **304 Product or plant local name** | **305 Disease condition(s) taken for/treated** | **306 When taken (month of pregnancy)** | **307 Duration of use (number of days)** |
| --- | --- | --- | --- |
| Garlic/nech shinkurt for 1,2,4,5/13 | 1. cough  2. common cold  3. stomachache/gastritis  4. nausea and vomiting  5. abdominal pain  6. diarrhea  7. wound  8. for preparation for birth difficulty  9. febrile illnesses/fever, chills, rigors, headache, cough, vomiting, loss of appetite, myalgia, arthralgia/  10. cough and common cold  11. cough, common cold, stomachache/ gastritis  12. nausea and vomiting, abdominal pain, diarrhea  13. cough, common cold, nausea and vomiting, abdominal pain  14. others_________ |  | 1. Only once  2. Occasionally  3. Weekly  4. Twice or more per week  5. Daily |
| Tenaadam for 1, 2, 3/11 |  |  |  |
| Nech bahir zaf for 1,2,3/11 |  |  |  |
| Damakase for pneumonia….9 |  |  |  |
| Hareg ressa for pneumonia…9 |  |  |  |
| Feto for 4, 5,6/12 |  |  |  |
| Girawa for 4, 5,6/12 |  |  |  |
| Enqoqo for 4,5/12 |  |  |  |
| Bunna/Gomen for wound…7 |  |  |  |
| Amoch ….8 |  |  |  |
| Tikur- azmud for 1,2/10 |  |  |  |
| Yemidir Embuway…1,2/10 |  |  |  |
| Gorteb for wound…7 |  |  |  |
|  |  |  |  |
|  |  |  |  |
|  |  |  |  |

| Part IV: **Herb medicines Sources and Reasons for its Use and non use** | | | |
| --- | --- | --- | --- |
| 401 | Who recommended the use of herbal preparations to you? **Please tick all that apply** | 1. My own idea  2. Family  3. Friends or neighbors  4. Traditional healers  5. Magazine or on social media  6. Doctor or pharmacist or other HP  7. Others, please specify |  |
| 402 | What is the sources/places you use to obtain herbal medicine: **Please tick all that apply** | 1. Self-preparation  2. Traditional healers or herbalists  3. Work places  4. Market places  5. Worship places  6. Family and/or friends |  |
| 403 | Did you inform your doctor/nurse/pharmacist about your use of herbal medicines during pregnancy? | 1. Yes  2. No | If no |
| 404 | Reason of non-disclosure | 1. Forgot it  2. Health professional did not ask me  3. Easy for use  4. It was not important  5. Afraid of health profession’s response  6. I did not visit the facility at the time of use |  |
| 405 | If you want information about herbal medicines, who would be your primary source of information? | 1. Health professionals  2. Traditional healers or herbalists  3. Religious leaders  4. Family or friends  5. Other, please specify |  |
| 406 | Please indicate the reasons(s) behind your use of herbal medicines during pregnancy: **Please tick all that apply** | 1. Herbal medicines are more effective than conventional medicines  2. Herbal medicines are safe in pregnancy  3. Herbal medicines are much cheaper than conventional medicines  4. Mistrust of conventional medicines  5. It is in my culture to use herbal medicines  6. I use herbal medicine when conventional medicine fails  7. Herbal medicines are more accessible than conventional medicines  8. Others, please specify: ---------------------- |  |
| 407 | Have you faced the side effect with HM use | 1. yes  2. no |  |
| 408 | Satisfaction with herbal medicine use | 1. Satisfied  2. Average  3. Dissatisfied |  |
| 409 | If you are not using any herbal medicines ever what was your reason of not use | 1. did not sick during pregnancy  2. lack of belief in the benefit of herbal medicines  3. afraid the side effects  4. did not hear about herbal medicines  5. lack of availability |  |
| Part V: **Medical Conditions Experienced during Pregnancy and their Treatment** | | | |
| 501 | During your current pregnancy, did you experience any medical conditions? | 1. Yes  2. No | If yes |

If “Yes”, please complete the following table to indicate what other condition(s) you experienced and when, and how you treated it/them:

| **502 types of medical Condition** | **503 Month of pregnancy** | **504 Treatment(s), Pharmaceutical, herbal, or combination** |
| --- | --- | --- |
| 1. Nausea, vomiting or morning sickness |  | 1. Pharmaceutical/conventional  2. herbal  3. both |
| 2. Abdominal pain or indigestion |  | 1. Pharmaceutical  2. herbal  3. both |
| 3. Urinary tract infection |  | 1. Pharmaceutical  2. herbal  3. both |
| 4. Headache |  | 1. Pharmaceutical  2. herbal  3. both |
| 5. Common cold |  | 1. Pharmaceutical  2. herbal  3. both |
| 6. Malaria |  | 1. Pharmaceutical  2. herbal  3. both |
| 7. High blood pressure |  | 1. Pharmaceutical  2. herbal  3. both |
| 8. Cough |  | 1. Pharmaceutical  2. herbal  3. both |
| 9. Back pain |  | 1. Pharmaceutical  2. herbal  3. both |
| 10. Vaginal problems (i.e., itching, bleeding, abnormal discharge, infection) |  | 1. Pharmaceutical  2. herbal  3. both |
| 11. Others…………………… |  | 1. Pharmaceutical  2. herbal  3. both |

**Thank you very much for participating in the current study!**
